# In Vitro Antiviral Activity and Projection of Optimized Dosing Design of Hydroxychloroquine for the Treatment of Severe Acute Respiratory Syndrome Coronavirus 2 (SARS-CoV-2)

**DOI:** 10.1093/cid/ciaa237

**Published:** 2020-03-09

**Authors:** Xueting Yao, Fei Ye, Miao Zhang, Cheng Cui, Baoying Huang, Peihua Niu, Xu Liu, Li Zhao, Erdan Dong, Chunli Song, Siyan Zhan, Roujian Lu, Haiyan Li, Wenjie Tan, Dongyang Liu

**Affiliations:** 1 Drug Clinical Trial Center, Peking University Third Hospital, Beijing, China; 2 MHC Key Laboratory of Biosafety, National Institute for Viral Disease Control and Prevention, China CDC, Beijing, China; 3 Department of Cardiology and Institute of Vascular Medicine, Peking University Third Hospital, Beijing, China; 4 Department of Orthopedics, Peking University Third Hospital, Beijing, China; 5 Research Center of Clinical Epidemiology, Peking University Third Hospital, Beijing, China

**Keywords:** Chloroquine, Hydroxychloroquine, SARS-CoV-2

## Abstract

**Background:**

The Severe Acute Respiratory Syndrome Coronavirus 2 (SARS-CoV-2) first broke out in Wuhan (China) and subsequently spread worldwide. Chloroquine has been sporadically used in treating SARS-CoV-2 infection. Hydroxychloroquine shares the same mechanism of action as chloroquine, but its more tolerable safety profile makes it the preferred drug to treat malaria and autoimmune conditions. We propose that the immunomodulatory effect of hydroxychloroquine also may be useful in controlling the cytokine storm that occurs late-phase in critically ill SARS-CoV-2 infected patients. Currently, there is no evidence to support the use of hydroxychloroquine in SARS-CoV-2 infection.

**Methods:**

The pharmacological activity of chloroquine and hydroxychloroquine was tested using SARS-CoV-2 infected Vero cells. Physiologically-based pharmacokinetic models (PBPK) were implemented for both drugs separately by integrating their *in vitro* data. Using the PBPK models, hydroxychloroquine concentrations in lung fluid were simulated under 5 different dosing regimens to explore the most effective regimen whilst considering the drug’s safety profile.

**Results:**

Hydroxychloroquine (EC_50_=0.72 μM) was found to be more potent than chloroquine (EC_50_=5.47 μM) *in vitro*. Based on PBPK models results, a loading dose of 400 mg twice daily of hydroxychloroquine sulfate given orally, followed by a maintenance dose of 200 mg given twice daily for 4 days is recommended for SARS-CoV-2 infection, as it reached three times the potency of chloroquine phosphate when given 500 mg twice daily 5 days in advance.

**Conclusions:**

Hydroxychloroquine was found to be more potent than chloroquine to inhibit SARS-CoV-2 in vitro.

inhibit SARS-CoV-2 in vitro.

### INTRODUCTION

In December 2019 the outbreak of a novel coronavirus, Severe Acute Respiratory Syndrome Coronavirus 2 (SARS-CoV-2 or COVID-2019), was first reported in Wuhan, China. The outbreak has since rapidly spread to other provinces in mainland China, as well as other countries around the world. Currently the number of people diagnosed with SARS-CoV-2 infection is increasing by approximately 1000 cases a day. Unfortunately, to date, no drugs have approved by regulatory agencies for the treatment of SARS-CoV-2 infection.

Chloroquine is a widely used anti-malarial with immunomodulatory effects [1-5]. In a recent *in vitro* study chloroquine was found to inhibit the growth of SARS-CoV-2 *in vitro* [6]. This finding has been supported by clinical studies conducted in approximately one-hundred SARS-CoV-2 infected patients [7, 8].

Hydroxychloroquine is an analog of chloroquine that has fewer concerns about drug-drug interactions. In the previous SARS outbreak, hydroxychloroquine was reported to have anti-SARS-CoV activity *in vitro* [9]. This suggests that hydroxychloroquine may be a potential pharmacological agent for the treatment of COVID-19 infection. However, to date, there is no clinical evidence to support the use of hydroxychloroquine as a treatment for SARS-CoV-2 infection.

The molecular mechanism of action of chloroquine and hydroxychloroquine has not been fully elucidated. Findings from previous studies have suggested that chloroquine and hydroxychloroquine may inhibit the coronavirus through a series of steps. Firstly, the drugs can change the pH at the surface of the cell membrane and thus, inhibit the fusion of the virus to the cell membrane. It can also inhibit nucleic acid replication, glycosylation of viral proteins, virus assembly, new virus particle transport, virus release and other processes to achieve its antiviral effects [10].

A reliable estimation of hydroxychloroquine and chloroquine concentrations in the lung, the target tissue, may be used for guiding dose recommendations. Physiologically-based pharmacokinetic (PBPK) models are a mathematical modelling technique that can predict drug concentrations in human tissues *in silico* by integrating physiological and drug disposition parameters. PBPK models are widely used in drug development to help identify whether a clinical trial is warranted as well as help guide the use of drugs based on predictions from well-validated models [11, 12].

In this study we aimed to: (i) investigate the antiviral and prophylactic activity of hydroxychloroquine and chloroquine *in vitro*, (ii) build a PBPK model for hydroxychloroquine and chloroquine using data from literature, and, (iii) predict drug concentrations under different dosing regimens using the developed PBPK models.

### METHODS

#### *In Vitro* Antiviral Activity Experiment

#### Experiment Materials

Chloroquine phosphate and hydroxychloroquine sulfate were purchased from Beijing Innochem Science & Technology Co, Ltd. The lyophilized powder was diluted in double distilled water to 10 mM. Hydroxychloroquine sulfate was readily soluble in water. Chloroquine phosphate was dissolved by shaking the solution at room temperature for 2 hours. The chloroquine and hydroxychloroquine solutions were filtered through a 0.22 μm membrane and were then stored at −80°C. The clinically isolated SARS-CoV-2 virus strain, C-Tan-nCoV Wuhan strain 01, was propagated in Vero cells.

**Cell Culture**

The Vero cells were derived from the African green monkey kidney and were grown in Dulbecco's Modified Eagle Medium (DMEM) (Sigma Aldrich, Boston, MA, USA) supplemented with 5% fetal bovine serum (Logan, UT, USA). The cells were maintained in a humidified atmosphere with 5% $CO_2$ at 37°C. The culture medium was replaced each day.

**Antiviral Activity Assay**

The anti-SARS-CoV-2 activity of chloroquine and hydroxychloroquine was investigated *in vitro*. Cells were seeded into 96-well plates at a density of $1\times10^4$ cells/well and were grown for 24 hours. The *in vitro* experiment was divided into two sections, named: (i) the treatment study and (ii) the prophylactic study.

Treatment study: In the treatment study Vero cells were infected at a multiplicity of infection (MOI) of 0.01 (100 PFU/well) for 2 hours at a temperature of 37°C .Virus input was washed with DMEM and the cells were then treated with medium containing either chloroquine or hydroxychloroquine at 0.032, 0.16, 0.80, 4, 20, 100 μM for 24 or 48 hours.

Drug pretreatment study: Vero cells were pretreated with chloroquine or hydroxychloroquine for 2 hours and then, were removed from the drug-containing medium. The virus-containing medium was then added to the infected Vero cells (as described for the treatment study) for 2 hours. Following this, the virus-containing medium was removed and replaced with fresh medium that did not contain drugs or viruses.

The supernatant was collected, and, the RNA was extracted and analyzed by relative quantification using RT-PCR (methods described in a previously published study) [13, 14].

**Viral RNA Extraction and RT-PCR**

Viral RNA was extracted from 100 μL of supernatant of infected cells using the automated nucleic acid extraction system (TIANLONG, China) and the manufacturer's instructions. Detection of the SARS-CoV-2 virus was performed using the One Step Prime Script RT-PCR kit (TaKaRa, Japan) on the Light Cycler 480 Real-Time PCR system (Roche, Rotkreuz, Switzerland) with primers. The following sequences were used:

forward primer: 5′-AGAAGATTGGTTAGATGATGATAGT-3′; reverse primer:5′-TTCCATCTCTAATTGAGGTTGAACC-3′; and probe:5′-FAM-TCCTCACTGCCGTCTTGTTG ACCA-BHQ1-3′.

All experiments were conducted in triplicates. The relative expression was estimated using the $2^{-\triangle\triangle Ct}$ method.

**Statistical Analysis**

A sigmoidal concentration-response function, Y=Bottom + (Top-Bottom)/ (1+10^((LogEC$_{50}$-X)*HillSlope)), was fit to the data using nonlinear regression. The

$EC_{50}$ values were calculated using PRISM (GraphPad software, San Diego, CA, USA).

**PBPK Model Development, Validation and Simulation**

The PBPK models for chloroquine and hydroxychloroquine were developed using Simcyp simulator (version 18). The chloroquine compound file was provided by Simcyp Limited (a Certara company, Blades Enterprise Centre, Sheffield, UK) and the hydroxychloroquine compound file was self-built. Physical and chemical parameters were obtained from the literature [15]. Pharmacokinetic parameters, such as liver intrinsic clearance, fa and ka, were determined from clinical data [16]. These data are summarized in supplement 1. The lung to blood concentration ratio for chloroquine and hydroxychloroquine (obtained from animal studies) was used to predict the drug concentration in the lungs [17, 18].

**Validation Data**

Published chloroquine and hydroxychloroquine clinical trial data were used to validate the developed PBPK models (details summarized in supplement 2) [16, 19-23]. Data obtained from the literature in graphical form were extracted using Plot Digitizer (version 2.26, GetData). Pharmacokinetic parameters that could not be sourced from the literature were estimated using extracted data in Phoenix (version 8.6, Certara company).

**Validation Method**

Concentration-time profiles were simulated under different published clinical trial protocols using the developed PBPK models for hydroxychloroquine and chloroquine [16, 19-23]. The Simcyp "Healthy volunteer", "Chinese healthy volunteer" and "Pediatric" virtual populations were used in the simulations as the clinical trials were conducted in Caucasian, Chinese and children populations, respectively.

Simulated exposure data was compared to observed data. The criterion to determine model accuracy was based on whether the observed data fell within the 90% confidence interval of the predicted values. The ratio of predicted pharmacokinetic (PK) parameters (e.g. $C_{max}$ and AUC) to observed values was used to evaluate model performance. The predicted values were considered reasonable if the ratio of predicted to observed data was within a 2-fold range ($0.5 \leq ratio \leq 2.0$).

**Simulation Method**

The exposure of chloroquine and hydroxychloroquine in the lungs, plasma and blood were simulated under different dosing regimens (shown in Table 1) using the validated PBPK models. A correction factor for chloroquine base and hydroxychloroquine base was input into the model simulations. Chloroquine phosphate 500 mg is equivalent to 300 mg of chloroquine base and 200 mg of hydroxychloroquine sulfate is equivalent to 155 mg of hydroxychloroquine base. The "Chinese Healthy Volunteers" virtual population provided in Simcyp was used for the simulations. All simulations were performed with 10 trials and 10 subjects per trial. Virtual subjects were aged between 20 to 50 years of age, and, 50% of the subjects were male and 50% female.

### Dose regimen optimization

The PBPK models were used to predict the lung tissue concentrations of chloroquine and hydroxychloroquine under different dosing regimens (Table 1). The lung trough concentration on days 1, 3, 5 and 10 were adjusted by the plasma unbound fraction ($f_{u,plasma}$) to obtain the free lung trough concentration. The ratio of the free lung trough concentration to the *in vitro* $EC_{50}$ values ($R_{LTEC}$) was calculated and the results tabulated. In a recent clinical trial 500 mg of chloroquine phosphate given twice daily was shown to be effective on study day 5 ($R_{LTEC,\ day5}$). This dosing regimen for chloroquine was used as the target for dose optimization for hydroxychloroquine (i.e., the $R_{LTEC}$ of hydroxychloroquine should not be lower than the $R_{LTEC,\ day5}$ of chloroquine at any time).

## RESULTS

### Antiviral Activity *in vitro*

Results from the *in vitro* study showed that both chloroquine and hydroxychloroquine have good antiviral activity. Chloroquine and hydroxychloroquine were found to decrease the viral replication in a concentration-dependent manner. The $EC_{50}$ values for chloroquine were 23.90 and 5.47 μM at 24 and 48 hours, respectively (Figure 1a). $EC_{50}$ values for hydroxychloroquine were 6.14 and 0.72 μM at 24 and 48 hours, respectively (Figure 1b).

### Antiviral Pre-treatment Activity *in vitro*

Hydroxychloroquine exhibited a superior *in vitro* antiviral effect in comparison to chloroquine when drug was added prior to the viral challenge. The $EC_{50}$ values for chloroquine were >100 and 18.01 μM at 24 and 48 hours, respectively. $EC_{50}$ values for hydroxychloroquine were 6.25 and 5.85 μM at 24 and 48 hours, respectively. It was noted that with longer incubation times the $EC_{50}$ values for chloroquine and hydroxychloroquine tended to decrease. The inhibitory effect of chloroquine was poor. This was particularly evident at 24 hours, whereby, even at the highest concentration of chloroquine used in the study, the inhibition rate did not exceed 50% (Figure 1c and 1d).

**PBPK model development, validation and simulation**

**Validation results**

The predicted and observed plasma/blood concentration time profiles for chloroquine and hydroxychloroquine is shown in Figure 2. Intravenous data was used to understand the distribution and elimination phase of the two drugs, and, oral administration data was used to understand the intracorporal absorption process. Most of the observed data fell within the 90% prediction interval. The ratio of predicted to observed PK parameters ($C_{max}$ and AUC) were within the range of 0.5 to 2.0 (details summarized in supplement 2), indicating that the prediction accuracy of the developed PBPK models was acceptable and could be used to simulate the different dosing scenarios.

**Simulation results**

The simulated lung, blood and plasma concentration time profiles for chloroquine and hydroxychloroquine under the different dosing regimens is shown in Figure 3. It can be seen that the lung, blood and plasma concentrations of chloroquine increased slowly after the first dose was given and was yet to reach steady state on day 10. The simulated chloroquine concentration in lung tissue was much higher than in plasma, where the lung to plasma ratio increased with time and reached a ratio of approximately 400. The projected lung, blood and plasma concentrations of hydroxychloroquine rapidly increased and reached steady state following the initial loading dose and subsequent maintenance doses (Figure 3b to 3f).

**Suggested dosing regimens for hydroxychloroquine to treat SARS-CoV-2 infection**

The free lung trough concentrations were also projected from the simulations. The ratio of free lung trough concentration to $EC_{50}$ ($R_{LTEC}$) under the different dosing regimens is shown in Table 1. The $R_{LTEC}$ values of hydroxychloroquine were found to be higher than the $R_{LTEC}$ values of chloroquine on days 1, 3, 5 and 10. This suggests that hydroxychloroquine may achieve ideal clinical efficacy under the simulated dosing regimens.

The $R_{LTEC}$ on day 1 was notably higher for hydroxychloroquine than for chloroquine. This is likely to be due to the loading dose of hydroxychloroquine given, thus enabling a faster clinical effect. There was no significant difference between the once and twice daily maintenance dosing regimens (Regimen C and D, respectively) when used from day 2 to day 10; hence, the once daily dosing regimen may be preferred to improve patient compliance. Despite Regimen F being a 5 day treatment regimen, the lung trough concentrations were still above the target concentration on day 10. However, if the treatment duration of Regimen F was extended to 10 days (i.e. Regimen E) it resulted in a higher drug concentration on day 10. Overall, Regimen F may be the best regimen whilst considering both efficacy, safety and patient compliance.

**DISCUSSION**

In this study, hydroxychloroquine exhibited better *in vitro* anti-SARS-CoV-2 activity than chloroquine. This was demonstrated by the $EC_{50}$ values for hydroxychloroquine always being smaller than the $EC_{50}$ values for chloroquine, indicating that hydroxychloroquine has a more potent antiviral activity (shown in Figure 1). In the study by Wang et al [6], chloroquine was shown to have an inhibitory effect on SARS-CoV-2 with an $EC_{50}$ value of 1.13 μM after a 48 hour incubation time. These findings are comparable with our *in vitro* chloroquine results of an $EC_{50}$ value of 5.47 μM. In addition, an unpublished clinical trial has demonstrated the therapeutic effect of chloroquine in SARS-CoV-2 infected patients. On the basis of hydroxychloroquine’s superior antiviral and prophylactic activity, as well as its more tolerable safety profile in comparison to chloroquine, we believe that hydroxychloroquine may be a promising drug for the treatment of SARS-CoV-2 infection [24].

In our study we noted that the $EC_{50}$ values for hydroxychloroquine and chloroquine decreased with longer incubation times. This suggests that incubation time may influence the drug's antiviral activity. Both hydroxychloroquine and chloroquine have been reported to accumulate in cells [25]. It is possible that a longer incubation time may provide more time for the drug to accumulate to higher intracellular concentrations and ultimately exhibit a better antiviral effect [26]. Another possible explanation is that the drug-induced cytotoxicity may take time to develop, and hence, the drug effect may increase with time [27].

The PBPK model for hydroxychloroquine and chloroquine was validated with *in vivo* PK data from humans, rats and mice. The model was able to reasonably predict drug concentrations in human lung tissue. A permeability rate limiting model was implemented in the PBPK model to mimic the characteristics of both drugs. In addition, a high lung to plasma partition coefficient ratio (Kp ratio) was used to imitate the drugs' high accumulation in lung tissue. The Kp ratio for humans was assumed to be same as the ratio for rats because there was no human data available. This assumption may be reasonable as the transportation of both drugs is completely via passive diffusion (i.e. no transporters are involved).

In some patients it has been reported that their immune response to the SARS-CoV-2 virus results in the increase of cytokines IL-6 and IL-10 [13, 28]. This may progress to a cytokine storm, followed by multi-organ failure and potentially death. Both hydroxychloroquine and chloroquine have immunomodulatory effects and can suppress the increase of immune factors [29, 30]. Bearing this in mind, it is possible that early treatment with either of the drugs may help prevent the progression of the disease to a critical, life-threatening state. In critically ill SARS-CoV-2 infected patients the use of corticosteroids may be harmful [31]. Whilst, the use of immunosuppressants (e.g. tocilizumab) are not ideal either as it can suppress the immune system and lead to an increased risk of infection [32]. In this setting, hydroxychloroquine may be an ideal drug to treat SARS-CoV-2 infection as it can inhibit the virus via its antiviral effects and help mediate the cytokine storm via its immunomodulatory effects. Based on work conducted in our lab, we recommend the concomitant use of low dose hydroxychloroquine with an anti-inflammatory drug to help mitigate the cytokine storm in critically ill SARS-CoV-2 patients.

Several clinical trials are currently investigating the use of hydroxychloroquine to treat SARS-CoV-2 infection. However, it is worth noting that the dosing regimens used in these trials are mainly based on previous clinical experience, raising the concern that adverse effects may occur in study participants (supplement 3). In this study, an optimized dosing regimen was designed for hydroxychloroquine to have a high loading dose and low maintenance dose based on it's unique pharmacokinetics (i.e. high accumulation in cells and long elimination half-life). Using PBPK modelling and simulation techniques the optimal dosing regimen for hydroxychloroquine was evaluated in *in silico*. The simulation results demonstrated that Regimen F was able to achieve treatment efficacy as well as have a good safety profile, even considering possible underestimation of drug efficacy to some extent. However, future clinical trials evaluating this regimen are required before it can be readily used in the clinic. The combination of the *in vitro* antiviral activity data and predicted drug concentrations in this study are being used to support the design of dosing regimens used in a future clinical trial.

### Acknowledgements

We want to thank Dr. Lisa Almond and Luke Guilliatt from Simcyp Limited for providing the chloroquine compound file.

### Funding

This work was supported by the “13th Five-Year” National Major New Drug Projects of China, Ministry of Science and Technology of the People’s Republic of China, [grant number No.2017ZX09101001-002-001] and Bill & Melinda Gates Foundation [OPP1204780].

### Conflict of Interest

C.S., H.L., and D.L. have patents pending for Anti-microbial infection pharmaceutical composition and its application. All other authors have no potential conflicts.

### Figures

Figure 1. The antiviral activities of chloroquine and hydroxychloroquine for treatment or prophylactic treatment against SARS-CoV-2 in vitro. The antiviral activities of chloroquine and hydroxychloroquine for therapeutic and prophylactic use were tested on the Vero cells infected with SARS-CoV-2 clinically isolate strain. (a) and (b): For treatment group, chloroquine and hydroxychloroquine were added medium after the Vero cells infected and cells were incubated with medium contained drugs for 24 or 48 hours. (c) and (d): For prophylactic treatment group, the Vero cells were pre-treated with chloroquine and hydroxychloroquine for 2 hours, and then viruses were added to medium to infect cells. After that, the medium was removed and replaced with fresh medium without drugs or viruses and cells were incubated for 24 or 48 hours. The viral yield in the cell supernatant was quantified by RT-PCR.

Figure 2. Predicted and observed mean arithmetic concentration profiles. (a) and (b): validation for hydroxychloroquine (HCQ) PBPK model by blood data after intravenous administration; (c): validation for HCQ PBPK model by blood data after oral administration; (d): validation for HCQ PBPK model by plasma data after oral administration; (e): validation for chloroquine (CQ) PBPK model by blood data after oral administration; (f): validation for CQ PBPK model by blood data after intravenous administration; (g): validation for CQ PBPK model by plasma data after oral administration; (h): validation for CQ PBPK model by plasma data after oral administration. Details were summarized in supplement 2.

Figure 3. Predicted plasma (a), blood (b), and lung (c) concentration-time profiles of chloroquine (CQ) under the dose regimen A, and hydroxychloroquine (HCQ) under dose regimen B, regimen C, regimen D, regimen E, and regimen F.

### Tables

**Table 1: Ratios of free lung tissue trough concentration/$EC_{50}$ ($R_{LTEC}$) under different dosage regimens**

| Drug | NO. | Dosing Regimen | $R_{LTEC}$ | | | |
|---|---|---|---|---|---|---|
| | | | **Day1** | **Day3** | **Day5** | **Day10** |
| Chloroquine phosphate | A. | D1-D10 500 mg BID | 2.38 | 5.92 | 18.9 | 40.7 |
| Hydroxychloroquine sulfate | B. | D1 800 mg+400 mg；D2-D10 400 mg QD | 33.3 | 55.1 | 103 | 168 |
| | C. | D1 600 mg BID；D2-D10 400 mg QD | 31.7 | 54.7 | 103 | 169 |
| | D. | D1 600 mg BID；D2-D10 200 mg BID | 31.7 | 53.1 | 101 | 167 |
| | E. | D1 400 mg BID；D2-D10 200 mg BID | 21.0 | 38.9 | 85.4 | 154 |
| | F. | D1 400 mg BID；D2-D5 200 mg BID | 21.0 | 38.9 | 85.4 | 83.3 |

$R_{LTEC}$: ratio of free lung tissue trough concentration/$EC_{50}$.

Figure 1

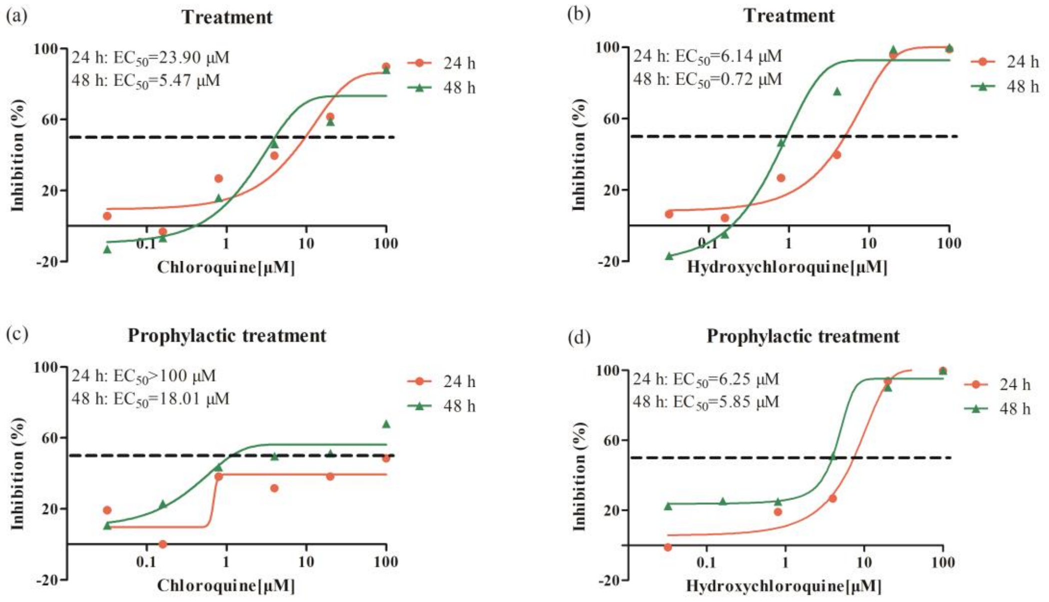

(a)
Treatment
24 h: EC50=23.90 μM
48 h: EC50=5.47 μM
24 h
48 h
Inhibition (%)
Chloroquine[μM]
(b)
Treatment
24 h: EC50=6.14 μM
48 h: EC50=0.72 μM
24 h
48 h
Inhibition (%)
Hydroxychloroquine[μM]
(c)
Prophylactic treatment
24 h: EC50>100 μM
48 h: EC50=18.01 μM
24 h
48 h
Inhibition (%)
Chloroquine[μM]
(d)
Prophylactic treatment
24 h: EC50=6.25 μM
48 h: EC50=5.85 μM
24 h
48 h
Inhibition (%)
Hydroxychloroquine[μM]

Figure 2

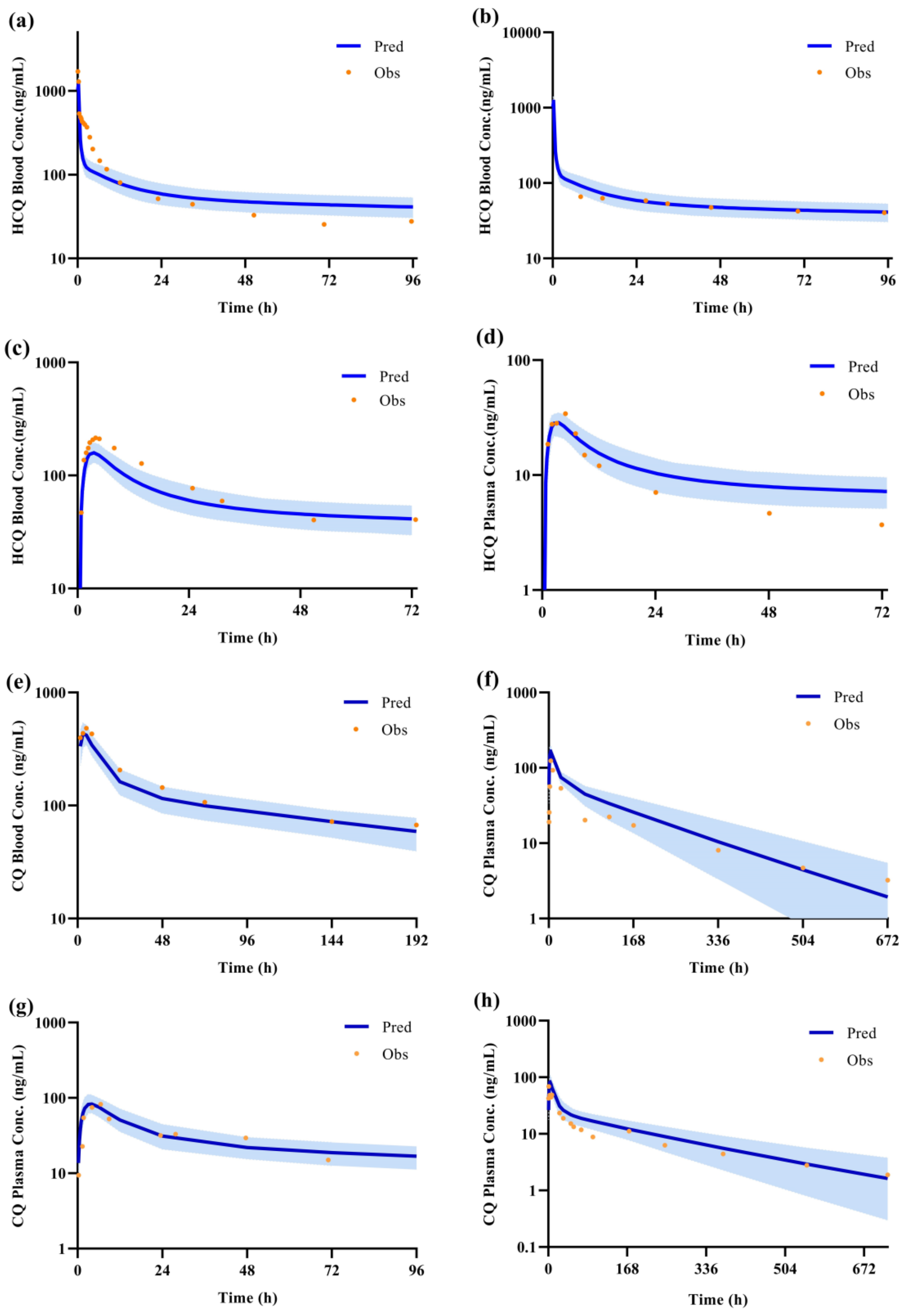

(a)
HCQ Blood Conc.(ng/mL)
Time (h)
Pred
Obs
(b)
HCQ Blood Conc.(ng/mL)
Time (h)
Pred
Obs
(c)
HCQ Blood Conc.(ng/mL)
Time (h)
Pred
Obs
(d)
HCQ Plasma Conc.(ng/mL)
Time (h)
Pred
Obs
(e)
CQ Blood Conc. (ng/mL)
Time (h)
Pred
Obs
(f)
CQ Plasma Conc. (ng/mL)
Time (h)
Pred
Obs
(g)
CQ Plasma Conc. (ng/mL)
Time (h)
Pred
Obs
(h)
CQ Plasma Conc. (ng/mL)
Time (h)
Pred
Obs

Figure 3

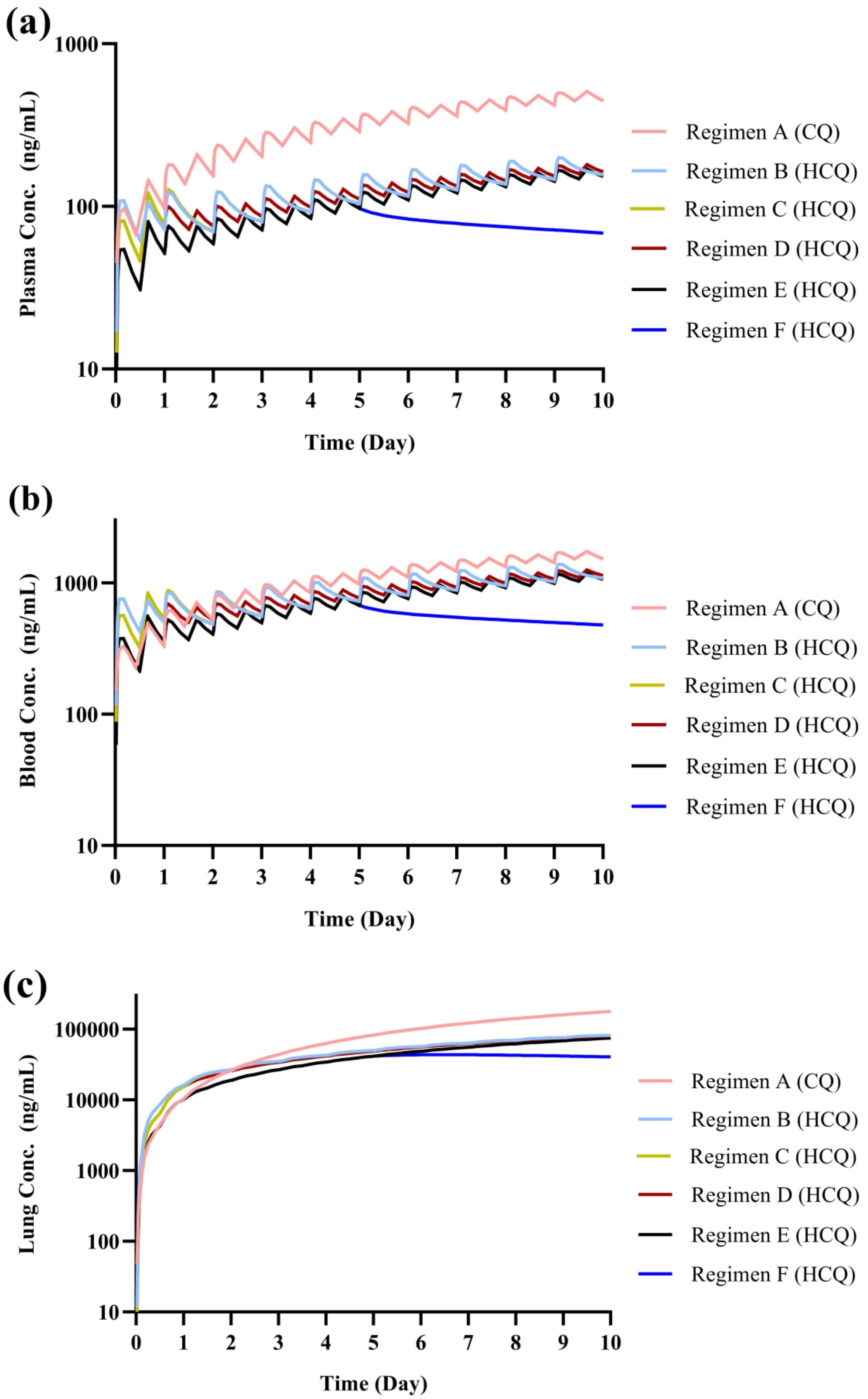

(a)
1000
100
10
Plasma Conc. (ng/mL)
0 1 2 3 4 5 6 7 8 9 10
Time (Day)
Regimen A (CQ)
Regimen B (HCQ)
Regimen C (HCQ)
Regimen D (HCQ)
Regimen E (HCQ)
Regimen F (HCQ)
(b)
1000
100
10
Blood Conc. (ng/mL)
0 1 2 3 4 5 6 7 8 9 10
Time (Day)
Regimen A (CQ)
Regimen B (HCQ)
Regimen C (HCQ)
Regimen D (HCQ)
Regimen E (HCQ)
Regimen F (HCQ)
(c)
100000
10000
1000
100
10
Lung Conc. (ng/mL)
0 1 2 3 4 5 6 7 8 9 10
Time (Day)
Regimen A (CQ)
Regimen B (HCQ)
Regimen C (HCQ)
Regimen D (HCQ)
Regimen E (HCQ)
Regimen F (HCQ)
